# Study of Ecophysiological Responses of the Antarctic Fruticose Lichen *Cladonia borealis* Using the PAM Fluorescence System under Natural and Laboratory Conditions

**DOI:** 10.3390/plants9010085

**Published:** 2020-01-09

**Authors:** Sung Mi Cho, Hyoungseok Lee, Soon Gyu Hong, Jungeun Lee

**Affiliations:** 1 Unit of Research for Practical Application, Korea Polar Research Institute, Incheon 21990, Korea; smcho@kopri.re.kr; 2 Division of Polar Life Sciences, Korea Polar Research Institute, Incheon 21990, Korea; soulaid@kopri.re.kr (H.L.); polypore@kopri.re.kr (S.G.H.); 3 Polar Sciences, University of Science and Technology, Daejeon 34114, Korea

**Keywords:** fruticose lichens, *Cladonia borealis*, Antarctic, phytochemistry, poikilohydric, non-photochemical quenching, desiccated state, shade-adapted lichen

## Abstract

Antarctic lichens have been used as indicators of climate change for decades, but only a few species have been studied. We assessed the photosynthetic performance of the fruticose lichen *Cladonia borealis* under natural and laboratory conditions using the PAM fluorescence system. Compared to that of sun-adapted *Usnea* sp., the photosynthetic performance of *C. borealis* exhibits shade-adapted lichen features, and its chlorophyll fluorescence does not occur during dry days without rain. To understand its desiccation-rehydration responses, we measured changes in the PSII photochemistry in *C. borealis* under the average light intensity of dawn light and daylight and the desiccating conditions of its natural microclimate. Interestingly, samples under daylight and rapid-desiccation conditions showed a delayed reduction in *Fv*’/*Fm*’ and rETRmax, and an increase in Y(II) and Y(NPQ) levels. These results suggest that the photoprotective mechanism of *C. borealis* depends on sunlight and becomes more efficient with improved desiccation tolerance. Amplicon sequencing revealed that the major photobiont of *C. borealis* was *Asterochloris irregularis*, which has not been reported in Antarctica before. Collectively, these results from both field and laboratory could provide a better understanding of specific ecophysiological responses of shade-adapted lichens in the Antarctic region.

## 1. Introduction

Antarctic climates are extremely cold, with high irradiation and strong winds, and therefore, the photosynthetic organisms in this area experience repeated desiccation-rehydration conditions [1,2,3]. Antarctic vegetation is mainly composed of the poikilohydric organisms, mosses and lichens [4,5]. Poikilohydry is accompanied by the ability to survive in a desiccated state (below 10% relative humidity) for long periods [1,2,3], which allows these organisms to inhabit extreme environments, such as desert, the Arctic, and Antarctic ecosystems where vascular plants fail to adapt [6,7,8]. 

Two mechanisms have been reported for desiccation tolerance in photosynthetic organisms, which are a delay in the rate of water loss and rapid repair after desiccation [9]. The former, which usually occurs in vascular plants, extends the time required for dehydration by days or even weeks and focuses on protecting against desiccation damage [10,11]. In contrast, the latter operates in nonvascular plants including algae, bryophytes, and lichens, and works efficiently to restore respiration and photosynthesis within a few minutes after rapid desiccation (RD) of less than 1 h [9,12]. As prolonged desiccation-rehydration cycles concomitantly produce reactive oxygen species (ROS) in the chloroplast and mitochondria, photobionts especially must also develop photoprotective machinery [13,14]. Thermal dissipation during desiccation is an effective photoprotection mechanism because the rate of heat dissipation is faster than its usage for photochemistry in the reaction center or for fluorescence emission [15,16,17]. Thermal dissipation progress called ‘non-photochemical quenching (NPQ)’ is known to prevent, to some extent, ROS formation induced by photooxidative damage using different carotenoids and the reversible xanthophyll cycle.

As lichens are complex symbiotic organisms consisting mainly of mycobionts (fungi) and photobionts (algae and/or cyanobacteria), the NPQ response of each lichen species could also vary due to interactions between the mycobionts and photobionts and/or others symbiotic organisms. Indeed, the NPQ responses of several lichens are differentially regulated by solar irradiation (intensity and duration) and the extent of desiccation. A seasonal variation study revealed that NPQ positively associates with solar irradiation in *Lobaria pulmonaria* [18,19]. In other cases, NPQ was shown to increase when the water content was reduced in *Parmelia quercina* [20], or it was maintained in *Ramalina maciformis* and *Cladonia vulcani* via a de-epoxidation reaction of the xanthophyll cycle under desiccation [21,22]. Although it is necessary to understand physiological responses during the NPQ process at the molecular level, the previously mentioned studies have indicated that lichens can have species-specific physiological responses, which probably are adaptations to their habitat. 

In recent decades, lichens have been widely used in environmental monitoring, especially to study the effect of air pollution; however, their role as a bio-indicator reflecting climate changes in the Antarctic region was recently reassessed [23,24]. Approximately 400 species of lichens reside on dry and rocky surfaces rather than in wet habitats in the Antarctic Peninsula [24]. Members of the lichen genus *Usnea* are distributed across most dry habitats of Antarctica and have been used to investigate ecophysiology, antioxidant production, and photoinhibition responses and in long-term and short-term monitoring studies [23,24,25,26,27,28]. In *U. antarctica*, for example, their growth rate is strongly correlated with mean summer temperature changes, and photoinhibitory quenching (qI)—a component of NPQ—mainly works to protect photosystems from high light intensity [23,24,26,27]. In addition, a low NPQ response is consistent with the finding that a small amount of glutathione is induced by high light levels, indicating that *U. antarctica* is likely to produce secondary compounds to protect against excess light [28].

Antarctic *Usnea* species have been extensively studied for several decades and are widely accepted as a model of Antarctic lichens. However, to understand the effects of climate change over the years, it is necessary to find other fruticose lichens with more sensitive photosynthetic performance. One candidate is *Cladonia*, a fruticose lichen genus, with 11 species on King George Island and 27 species reported throughout Antarctica [29,30,31,32]. *Cladonia borealis* S. Stenroos is found in the polar regions of both hemispheres and the alpine regions of the Andes Mountains [31,33,34], and it is one of the dominant species of Antarctic vegetation [35,36,37]. To date, studies on *C. borealis* have focused on its taxonomy, the genetic recognition of mycobiont and photobiont diversity, evolutionary history, and geographical distribution [35,37,38,39]. However, there have been no ecophysiological studies on this species focusing on its photosynthetic response to microclimate changes. 

In this study, we assessed the photosynthetic performance of *C. borealis* compared to that of *Usnea* sp. in response to microclimate changes under field conditions. In addition, to understand how *C. borealis* responds to desiccation-rehydration, we examined changes in PSII photochemistry under controlled laboratory conditions. We also analyzed major photobionts of this lichenized fungus by amplicon sequencing. We used the results of field and laboratory experiments to discuss the utility of *C. borealis* for biomonitoring the effects of climate change in the Antarctic region.

## 2. Results and Discussion

### 2.1. Site Description and Lichen Identification

**Figure 1:**
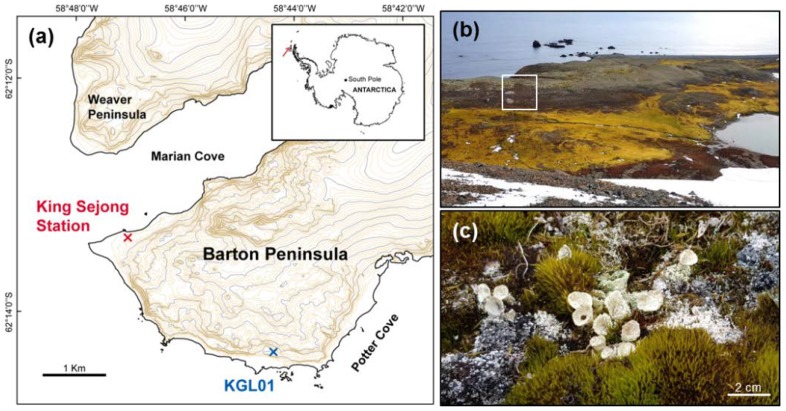
Location and landscape of the study site (KGL01: 62°14′24″ S, 58°44′36″ W), and an image of *C. borealis* at the field. (**a**) KGL01 on the map, placed near Potter Cove in Barton Peninsula. (**b**) This site has well-distinguished vegetation that consists of mosses and lichens. The area spanning the brown and grey parts was composed of lichens, and our observation was performed at the area indicated by the white box. (**c**) Several thalli of *C. borealis* accompanied by the moss *Chorisdontium aciphyllum* are shown.

The study site (KGL01: 62°14′24″ S, 58°44′36″ W, at an altitude of 39 m) is a windy hill front at the seashore near Potter Cove in Barton Peninsula, King George Island (Figure 1a). The mean annual temperature and rainfall are −1.1 °C and 42.8 mm, respectively. In the summer season from December to February, the average temperature is 2.1 °C with an average accumulated rainfall of 27.3 mm, which is lower than the annual average rainfall (Figure S1). The site is an area where various lichens and moss species appear and are supplied with water from melted snow, rainfall, or dewfall. This area also appears to have different vegetation depending on the water gradient generated by a stream. The vegetation around the resulting pond can be divided into two parts based on color (Figure 1b). The golden and brown parts, which appear closer to the pond, are areas dominated by mosses—*Sanionia uncinata* and *Chorisdontium aciphyllum*, which grow in a carpet-shape—and lichens consisting of the genera *Cladonia*, *Psoroma*, and *Ochrolechia* [35,37]. The grey part, the dry area, is largely covered by species of the genus *Usnea*. *C. borealis* is mainly distributed in the area spanning the brown and grey parts; the mature shape of this species has a stalk that is 3–5 cm tall and 1–2 cm in diameter, with an upper cortex that forms a curved cover (Figure 1c) [32,36]. 

**Figure 2:**
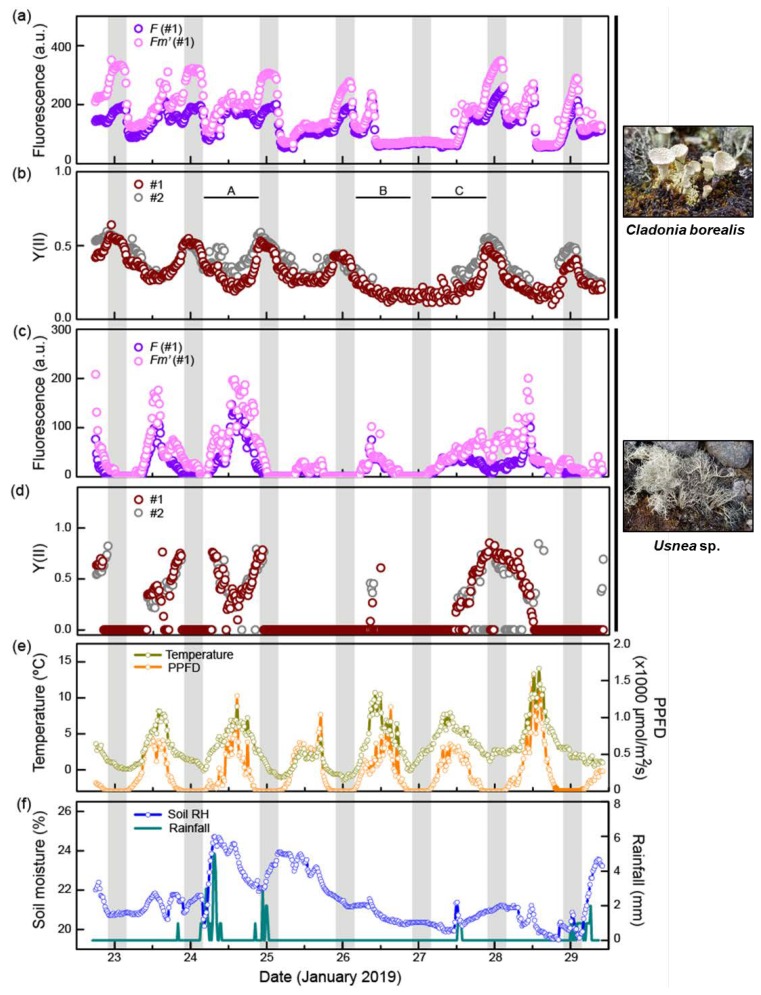
Field observation of the fluorescence (*F* and *Fm*’) and Y(II) value of *C. borealis* and *Usnea* sp. and microclimate at the site. Grey shading indicates nighttime (10 PM to 4 AM) when photosynthetic photon flux density (PPFD) was less than 10 μmol/m^2^/s. (**a**,**b**) Fluorescence and Y(II) value of *C. borealis* (**c**,**d**) Fluorescence and Y(II) value of *Usnea* sp. Data were obtained from two individual thalli (Samples #1, #2). Patterns of fluorescence (*F* and *Fm*’) of *C. borealis* and *Usnea* sp. were similar between the two samples. (**e**) Changes in temperature (°C) and PPFD (μmol/m^2^/s). (**f**) Changes in volumetric soil moisture (%) and rainfall (mm) during the observation period. *F*, current fluorescence; *Fm*’, maximum fluorescence.

The field survey to monitor the photosynthetic performance of *C. borealis* and the *Usnea* sp. was performed from 22–30 January 2019 (Figure 2). Microclimate factors such as photosynthetic photon flux (PPFD), air temperature (°C), rainfall (mm), and volumetric soil moisture (%) were also recorded. We identified our field samples as *C. borealis* based on the morphological and anatomical features described previously [36], and later confirmed by ITS sequencing. For lichen identification, fungal-specific ITS regions (~900 bp) were amplified, sequenced, and identified as *C. borealis* (NCBI accession: DQ534459) with 98% query coverage, an *E*-value = 0, and 99.3% identity.

### 2.2. C. borealis Photosynthetic Activity at Night and Dawn

Nighttime was identified by a PPFD value less than 10 μmol/m^2^/s; hence, the length of one night was approximately 6 h from 10 PM to 4 AM (Figure S2). During the observation period, temperature and PPFD changed with a diurnal rhythm. The temperature ranged from −1 °C to 14 °C and fell below 3 °C in the nighttime. Mean temperature during the daytime was 0.9 °C to 6.0 °C, and the coldest day was on 25 January (−1.1 °C to 4.3 °C). The mean daily PPFD value was generally 300–350 μmol/m^2^/s, and the strongest irradiation was recorded on 28 January, with a mean value of 466 μmol/m^2^/s and a maximum of 1595 μmol/m^2^/s (Table S1; Figure 2e). Volumetric soil moisture ranged from 19.5 to 24.7%. The mean value of soil moisture was higher, 21 to 23%, on three days (23–25 January), but less than 21% from 26–28 January. Accumulated rainfall was recorded every 10 min, and total daily rainfall varied randomly. The most rain fell on 24 January (60 mm), followed by 18 mm on 25 January; for all other days, there was no rain or rainfall was less than 10 mm (Table S1; Figure 2f). We were unable to obtain data for the relative humidity (RH) surrounding the lichens at that time; instead, we predicted the dryness based on records of both the volumetric soil moisture and rainfall events [40]. The fluorescence value represents the emission light intensity of chlorophyll a generated before (*F*) and after (*Fm*’) irradiation with a saturation pulse, and the difference between *F* and *Fm*’ directly correlates with the effective photosynthetic yield, Y(II): Y(II) = (*Fm*’ − *F*)/*Fm*’ (Figure 2a–d) [41]. At a glance, the photosynthetic yield of the *Usnea* sp. showed a significant correlation (R^2^ > 0.5) with PPFD and temperature, but not soil moisture; however, *C. borealis* showed less correlation (Table S2; Figure S3). In addition, the photosynthetic performance of *C. borealis* was quite different from that of the *Usnea* sp. 

First, the maximum Y(II) value of *C. borealis* (~0.6) was lower than that of the *Usnea* sp. (~0.8), but the inactivation time of Y(II) in *C. borealis* (12%, 57/491) was about six times shorter than that of the *Usnea* sp. (69%, 340/490). The inactivation state of photosynthesis occurs when there is no difference between *F* (present state) and *Fm*’ (maximum excitation capacity) values, meaning that the photosystems are not capturing photons. Inactivation occurs during photoinhibition or when the conditions for activation are insufficient. However, unlike the *Usnea* sp., the *C. borealis* photosynthetic performance was continuous and rhythmic in the Antarctic site (Figure 2). 

There was a remarkable difference between the photosynthetic performances of the *Usnea* sp. and *C. borealis* across the three times of day: dawn (4:00–6:00), daytime (6:00–22:00), and night (22:00–4:00) (Table 1). 

Photosynthetic activity of the *Usnea* sp. almost disappeared at night and dawn (~90% inactivation), and was highest during the daytime, where activation was only 58%. Photosynthetic performance of lichens generally increases 1–2 h after sunrise, and then rapidly decreases because high irradiation and an increase in temperature cause CO_2_ exchange to cease under the desiccating conditions [42]. Indeed, the growth rate of *Usnea antarctica* was sensitive to decreasing mean temperatures [23,24], and approximately 1000 μmol/m^2^/s light intensity was insufficient to induce photoinhibition [26,27]. Such photosynthetic features of *Usnea* sp. are probably associated with abundant inactivated states, which were observed in this study. In *C. borealis*, however, the mean Y(II) value was highest at night and gradually decreased from dawn to daytime. Low levels of inactivation were also observed at night and dawn (~7% inactivation) and daytime (~14% in Table 1).

Lichens often are distinguished by their responses to light intensity (typically classified as sun- or shade-adapted species), which is associated with their habitat and secondary compound production [43,44]. Sun-adapted lichen species show no inhibition under high light levels; instead, they produce a secondary compound to screen out the light [45,46], and *Usnea* sp. have been reported to have this characteristic [26,27,28]. In contrast, shade-adapted lichen species show photoinhibition to strong light irradiation, and their maximum photosynthetic activity exists before noon if they have not undergone water stress [40,47]. Taken together, the photosynthetic performance of *C. borealis* indicates that it can be vigorously active even at low temperatures and in the dim light conditions of dawn and nighttime, suggesting that it has features of a shade-adapted lichen.

### 2.3. Water Deficiency Is a Limiting Factor for Photosynthesis of C. borealis

**Figure 3:**
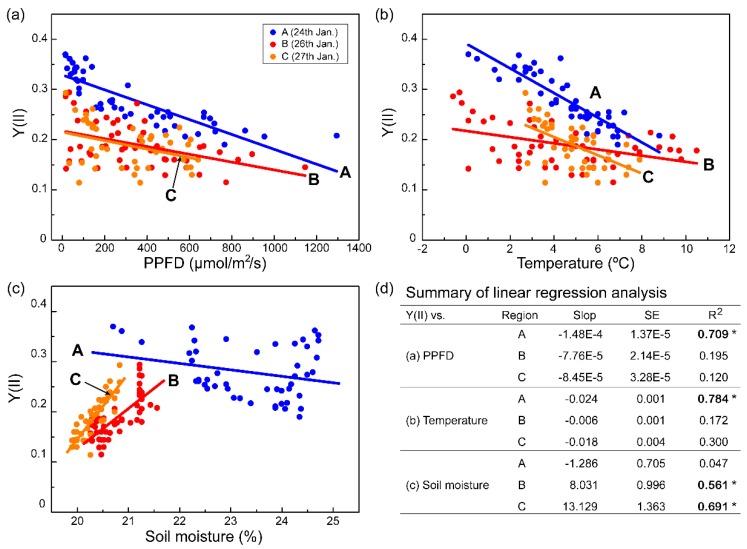
Correlation between Y(II) of *C. borealis* and PPFD (**a**), temperature (**b**), and volumetric soil moisture (**c**) parameters. Data were extracted from Regions A, B, and C in Figure 2b. A fitting analysis for linear regression was performed for each dataset. (**d**) The linear regression parameters are presented in a tabular format. Asterisks indicate R^2^ > 0.5. SE, standard error.

In this study, we successfully stimulated photosynthetic activity in response to microclimate changes in real time and showed how the photosynthetic efficiency of *C. borealis* is affected by microclimate changes. Interestingly, the Y(II) rhythm showed an irregular pattern in which night peaks were lost on the evening of 26 January, which was a dry day with low soil moisture; the most recent rainfall had been the night of 24 January. However, it rained the next afternoon, and the Y(II) had recovered by that night. To assess the effects of microclimate, we divided the observation period of Y(II) into three regions based on moisture conditions: wet (Region A, 24 January), dry (Region B, 26 January), and re-wetted (Region C, 27 January) (Figure 2b and Figure 3). In Region A, the Y(II) value was strongly negatively correlated with PPFD and temperature, but not with soil moisture. The Y(II) in Regions B and C, however, appeared to be influenced by the low level of soil moisture and not by the diurnal rhythms of PPFD or temperature (Figure 3). 

Although the low level of soil moisture was not directly related to the water content of *C. borealis*, the dramatic reduction in soil moisture and lack of rain from 25–26 January implied that the lichen was desiccated [40]. Furthermore, the strong correlation between Y(II) and soil moisture (R^2^ > 0.5) suggests that desiccation in *C. borealis* is a more limiting factor than high-light or low-temperature conditions. 

We also found that the fluorescence dynamics of both lichen species were remarkably different during desiccation (Figure 2). In the *Usnea* sp., the fluorescence (*F* and *Fm*’) value during two days of desiccation (25–26 January) was dramatically lower than on 24 January, which was rather humid, and the Y(II) was eventually inactivated. Besides, Y(II) was completely inactive on 25 January, presumably due to the average temperature being 4 °C. In *C. borealis*, however, Y(II) was active until the soil moisture was below ~21% at noon, indicating that *C. borealis* is more tolerant of desiccating conditions than the *Usnea* sp. From these observations, we expect that the photosystem II of *C. borealis* aggressively reacts to abiotic stresses in Antarctica using an effective photoinhibition process [48].

Lichens can absorb water from rain, fog, dew and air humidity [49,50,51], or substrate moisture [52], but, without rain, the water content of lichens is rapidly reduced via evaporation due to the high light intensity [40,51,53]. The fruticose form has the advantage of capturing water from the air because of its large surface area. However, the water retention ability differs depending on the specific lichen and its substrates. *C. borealis* thalli often grow close together, and they are commonly found on or near moss colonies. In contrast, *Usnea* species are usually found on rocks with spaces between them. Such substrate specificities and distribution patterns of thalli also greatly affect the acquisition of water from the air and the lichen’s water retention ability [40,54].

In this study, we used the Monitoring-PAM system, which allows high-throughput data acquisition for several days to months in the Antarctic field. This system provides chlorophyll fluorescence parameters (*F* and *Fm*’) in minute intervals. In contrast with previous reports that had measurements every few hours [45,55,56], this system provides real-time dynamics of photosynthetic performance in response to fluctuating environmental changes. From the data acquired in this study, we recognize that lichen species have different levels of photosynthetic performance sensitivity. Compared to the sun-adapted features of the *Usnea* sp., shade-adapted *C. borealis* has a highly susceptible photoinhibition process to protect against high light levels, low temperatures, and water-deficit conditions in its natural habitat. In addition, even small environmental changes can be a limiting factor in photosynthetic performance depending on the lichen species (Figure 2 and Figure 3). Taken together, these chlorophyll fluorescence monitoring data in real-time indicate that there are still unknown, lichen-species-dependent ecophysiological responses to microclimate changes in their niches.

### 2.4. Photoinhibition Response of C. borealis under a Laboratory Mimic of Microclimate Conditions

**Figure 4:**
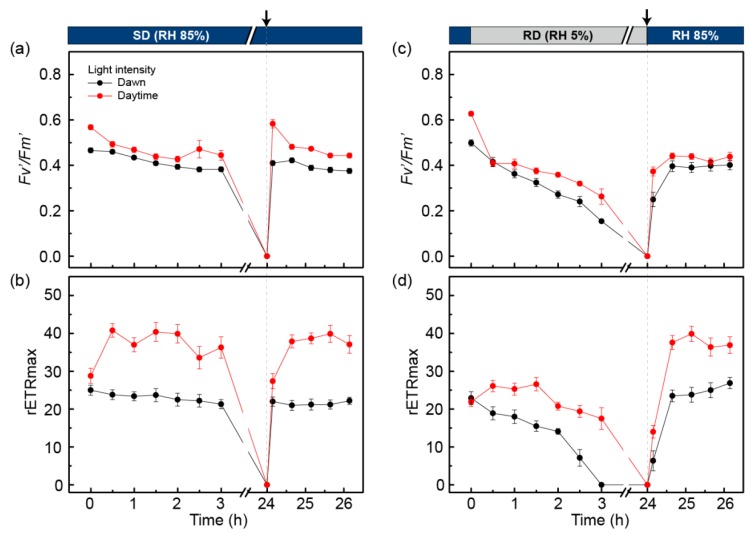
Changes in maximal photosynthetic yield (*Fv*’/*Fm*’) (**a**,**c**) and rETRmax (**b**,**d**) with dawn-light (50 μmol/m^2^/s) and daytime-light (220 μmol/m^2^/s) under a desiccation-rehydration cycle in *C. borealis*. Before desiccation, samples were activated with light (50 μmol/m^2^/s) for 2 h under fully hydrated conditions with distilled water. The thalli were treated under slow desiccation (SD, 85% RH) (**a**,**b**) and rapid desiccation (RD, 5% RH) (**c**,**d**) for 24 h (until indicated by the dashed line), and then rehydrated with a water spray of distilled water (black arrow). After the removal of excess water, both samples were kept under SD conditions for an additional 2 h. The ten biological replicates were used for each treatment (*n* = 10). Experiments were repeated at least two or three times using the same thalli after re-stabilizing at 50 μmol/m^2^/s light with an 18:6 light:dark cycle at 8 °C with hydration for a week. Results are the means with ± standard deviation shown by vertical bars.

The field monitoring results suggest that the photosynthetic performance of *C. borealis* is resistant to changes in light and temperature, but strongly downregulated under desiccating conditions (Region B in Figure 2); we, therefore, hypothesized that water availability is a limiting factor. Poikilohydric features have been known to provide lichens with strong tolerance to desiccation [1,2,3], but the desiccation-rehydration response of Antarctic *C. borealis* has not been investigated. We therefore first tested the poikilohydric response of *C. borealis*. We collected the thalli of *C. borealis* at the KGL01 site and measured the Y(II) value during air-drying (50% RH, 16 °C) in the laboratory at King Sejong Station, Barton Peninsula (Figure 1, Figure S4). Although the laboratory conditions were different from those in the field and the results may not be indicative of the situation in the field, we believe that this experiment indicates the time required to dry and rehydrate the lichen and whether it is poikilohydric. The relative water content (RWC) of the thalli constantly decreased over 3 h, while the Y(II) value almost persisted until ~20% of the RWC was achieved over 90 min of air-drying; thereafter, a rapid decline was observed (Figure S4). After 24 h of air-drying, the fluorescence value of *C. borealis* was no longer detected but it was dramatically recovered up to 90% after 10 min of rehydration with distilled water (Figure S4), confirming that *C. borealis* has this poikilohydric feature and a rapid repairing mechanism for desiccation damage [9,12,57]. The inactivation and reactivation of photosynthesis depending on the desiccation-rehydration cycle is believed to be a general phenotype of lichens observed in most terrestrial habitats, temperate regions, deserts, and the Arctic and Antarctic regions [6,8,55,56]. Next, to understand how *C. borealis* responds to desiccation-rehydration (Regions B and C in Figure 2), we performed a rapid light curve (RLC) experiment to reproduce the microclimate conditions of the species’ natural habitat (Figure 2); two light intensities, 50 μmol/m^2^/s (dawn light) and 220 μmol/m^2^/s (daytime light) were tested, with mild and severe desiccation conditions of 85% RH for slow desiccation (SD) and 5% RH for rapid desiccation (RD), respectively (Figure 4, Figure S5). 

Under SD conditions for 3 h, the maximal photosynthetic yield (*Fv*’/*Fm*’) and rETRmax of the dawn-light samples were slightly decreased to approximately 90% of their initial state (Figure 4a,b). After 24 h of treatment under SD conditions, fluorescence was not detected because the RWC of the samples was less than 10%. After 10 min of rehydration with distilled water, the *Fv*’/*Fm*’ and rETRmax were completely recovered to their initial state. For the daytime-light samples, the *Fv*’/*Fm*’ value gradually decreased to ~80% of the initial value during 3 h of treatment, while the rETRmax increased to 1.5-fold the initial value after 30 min of treatment. The values were then slightly decreased to ~90% of their maximums. After 10 min of rehydration, the *Fv*’/*Fm*’ and rETRmax of the daytime-light samples completely recovered to their initial state. These results indicate that the photosystem of *C. borealis* has SD resistance and a rapid repairing mechanism after rehydration. In addition, the increase in light intensity could help to improve the PSII efficiency and electron transfer rate in the photosystem. 

The RD conditions caused some oxidative damage in the photosystem of *C. borealis* compared to the samples treated with SD (Figure 4c,d). For the dawn-light samples treated for 3 h with RD, the *Fv*’/*Fm*’ value declined to ~20% of its initial state, and the rETRmax disappeared. After rehydration of the 24 h-dehydrated samples, *Fv*’/*Fm*’ was restored to ~80% of its initial value, and rETRmax completely recovered within 1 h of rehydration. For the daytime-light samples under RD conditions, *Fv*’/*Fm*’ was reduced to ~50% of its initial state after 3 h of treatment. In contrast, rETRmax was slightly increased (~1.1-fold) after a 30-min treatment but then decreased to ~80% of its initial state by 3 h. After rehydration of the 24 h-dehydrated daytime-light samples, the *Fv*’/*Fm*’ value reached ~80% of its initial value, similar to the rehydration response of the dawn-light samples. Such responses could imply that the increase in light intensity from dawn to daytime plays a positive role in enhancing PSII efficiency and electron transfer to PSI, just like the SD treatment. 

Interestingly, the rETRmax of the rehydration response was quite different from that of the RD-treated samples, which increased to twice the initial state, a value that is similar to that of the SD-treated samples (Figure 4d). From this result, we inferred that the RD conditions in this experiment could damage the oxygen-evolving center (OEC) of PSII but not the electron transfer process through plastoquinone (PQ) and PSI. In fact, the OEC component is easily exposed to ROS because it plays an important role in generating the electron gradient by charge separation from water molecules to electrons and oxygen molecules using light energy [42,48]. Furthermore, desiccation stress can severely affect the OEC component of PSII owing to the depletion of water molecules, and the photo-excited pigments easily produce ROS even under low-light conditions [58]. By sequential electron flow, oxidative pressure can damage PQ, which is the first molecule to be oxidized by P680+, and the PSI side as well [48]. Excessive or uncontrolled ROS levels are harmful to essential molecules, including nucleic acids, proteins, and lipids [42]. In *C. borealis*, however, the ETR reaction was completely rescued by rehydration, but the photosynthetic efficiency of PSII was not; therefore, we assume that the applied desiccation conditions damaged PSII, but not the PQ or PSI side. 

### 2.5. PSII Photochemistry Changes in C. borealis during the Dehydration Response

**Figure 5:**
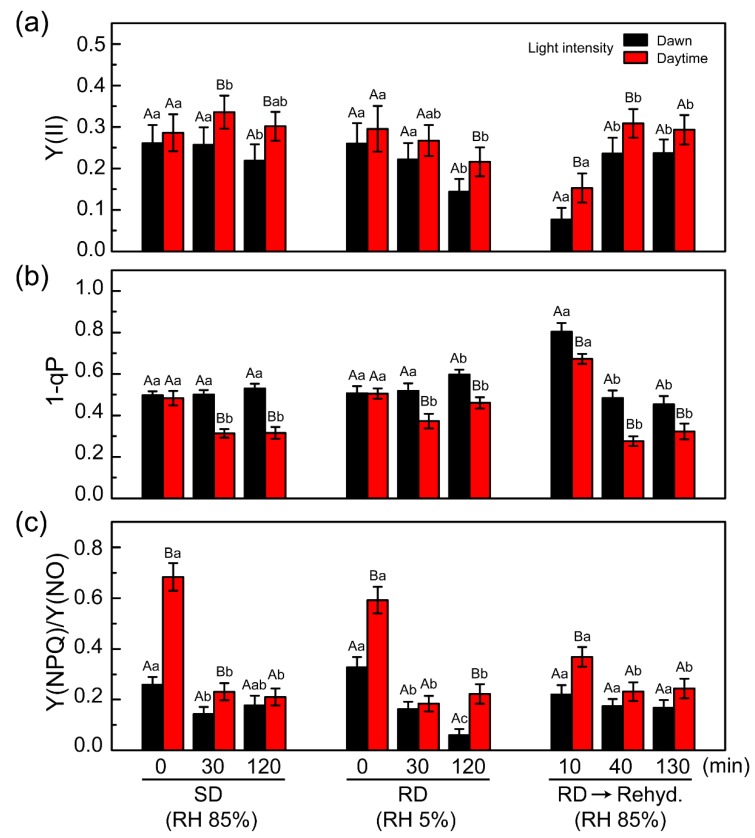
PSII photochemistry changes during RLC experiments under the given conditions. (**a**) Effective photosynthetic yield Y(II), (**b**) 1-qP value, and (**c**) Y(NPQ)/Y(NO) ratio. Each value indicates the average score ± standard deviation, calculated from the data in Figure S6. The average was calculated by two-way ANOVA, and its significant differences among the data were analyzed based on the Tukey’s HSD test (at *p* < 0.05) which was displayed with different letters; upper case letters indicate the effects of light intensity at the same time of desiccation (or rehydration) treatment, and lower case letters indicate the effects of desiccation (or rehydration) treatment at the same light condition. The ten biological replicates were used for each treatment (*n* = 10). Experiments were repeated at least two or three times using the same thalli after re-stabilizing at 50 μmol/m^2^/s light with an 18:6 light:dark cycle at 8 °C with hydration for a week. Black and red bars represent dawn light (50 μmol/m^2^/s) and daylight (220 μmol/m^2^/s), respectively. Y(NPQ), the efficiency of the regulated non-photochemical quenching reaction; Y(NO), the efficiency of the nonregulated non-photochemical quenching reaction; SD, slow desiccation; RD, rapid desiccation; RD→Rehyd., rehydration after 24 h of RD treatment.

The photosystem of *C. borealis* showed rapid repair via rehydration after SD and RD treatments. In particular, increased light intensity (50 to 220 μmol/m^2^/s) enhanced the recovery of PSII efficiency (*Fv*’/*Fm*’) and the electron transfer rate (Figure 4), and we, therefore, hypothesized that PSII protective mechanisms are induced by the changes in light intensity from dawn to daytime. From the RLC experiment, we could deduce how photosystem performance changed under saturated light conditions [59]. During SD, RD, and rehydration responses, the absorbed light energy can be used in a different manner depending on PSII status. Absorbed light energy can be competitively used for photochemistry at the reaction center of PSII, heat dissipation in NPQ, and re-emission of fluorescence. The efficiency of light energy usage can be calculated by the photochemical reaction, Y(II), the regulated non-photochemical reaction as heat dissipation, Y(NPQ), and the non-regulated non-photochemical reaction, Y(NO): Y(II) + Y(NPQ) + Y(NO) = 1 [60]. Thus, the PSII photochemistry changes in *C. borealis* under the given conditions indicate the photosystem response to a desiccation-rehydration cycle during the daytime (Figure 5). 

During daylight treatment, the Y(II) values were slightly higher than those of the dawn-light samples under SD, RD, and rehydration after RD exposure; however, the change in their patterns was similar (Figure 5a). The 1-qP value, which represents pressure of the PSII reaction center [61,62], was the same initially under SD and RD conditions, but this value was reduced in the daylight samples compared with that in the dawn light samples during treatment under both conditions (Figure 5b). This phenomenon could indicate that the PSII reaction center of the daylight samples was protected against induced pressure during treatment, after initiation. The rehydration response also showed that the dawn-light samples had slightly higher pressure than the daylight samples. This is consistent with the recovery rates of *Fv*’/*Fm*’ and rETR in the dawn-light samples, which were delayed relative to the values of the daylight samples (Figure 4).

Therefore, we suggest that the photosystems of *C. borealis* possess some protective mechanism(s) that are rapidly induced by daylight exposure (within 30 min), and these mechanisms can function to reduce pressure at the PSII reaction center. Consequently, these protective mechanisms likely associate with enhancement of PSII efficiency in the daylight samples, even under SD and RD conditions (Figure 4 and Figure 5). To clarify this possibility, the process had to be examined in more detail. 

One possibility for the daylight induced-photoprotective mechanism is an NPQ process in *C. borealis*. The Y(NPQ) and Y(NO) values represent the NPQ process, but the former is a regulated reaction that usually indicates heat dissipation by the xanthophyll cycle and some carotenoids, while the latter is a nonregulated reaction including the re-emission of light and photodamage [60]. The Y(NPQ)/Y(NO) ratio shows how excess light energy is used at the PSII reaction center. The Y(NPQ)/Y(NO) value of the daylight samples was 2-fold higher than that of the dawn-light samples at the start of SD and RD treatments (Figure 5c). Such a finding indicates that the NPQ process was further induced by daylight exposure, but after treatment initiation, it was stabilized under the SD and RD conditions. In general, the NPQ process has at least three components: pH-regulated energy dissipation in the antenna system of PSII, a state transition between PSII and PSI, and a photoinhibitory quenching process (qE, qT, and qI, respectively) [63,64,65,66]. These quenching processes have different reaction time scales, and qE usually has a fast phase of relaxation [67,68]. Our results could not determine which NPQ component plays a major role in the photoinhibition mechanism, but we speculate that qE could contribute to a rapid reduction in PSII pressure in *C. borealis*. This qE quenching process is activated by the enzymatic conversion of violaxanthin to zeaxanthin, called the xanthophyll cycle. The xanthophyll cycle is generally associated with desiccation tolerance in lichens [18,19,20,21,22]. For example, in *Lobaria pulmonaria*, the light-dependent xanthophyll cycle can enhance desiccation tolerance [69] and the generation of light-independent zeaxanthin plays an antioxidant role in stabilizing the membrane and downregulating photosynthetic efficiency [70].

This laboratory experiment could provide clues to understand the continuous and rhythmic photosynthetic performance of *C. borealis* in Antarctic environments. These changes in PSII photochemistry demonstrate that rapid NPQ induction by increased light intensity can enhance desiccation tolerance, and such phenomena are presumably associated with microclimate changes in the Antarctic region. As photosynthetic organisms in this area have a short growing season (December to February), they must create a balance between growth proliferation and a reduction in damage from environmental stressors. In the natural habitat, fluctuations in light, temperature, and humidity generate high pressure on PSII, resulting in excess ROS formation. Moreover, a lack of water molecules with sunlight exposure may overexcite the reaction center of PSII, regardless of low lighting [71], thereby resulting in a reduction in photosynthetic efficiency. To date, the photosynthetic performance of *C. borealis* has been shown to be highly susceptible to changes in environmental conditions, and we expect that such a shade-adapted lichen species has additional tightly regulated photoinhibition mechanisms owing to its complicated microclimate changes.

### 2.6. Major Photobiont of C. borealis Is Asterochloris Irregularis

Lichens are composed mainly of mycobionts (fungi) and photobionts (algae and/or cyanobacteria), but it was recently discovered that yeast can be involved as a third partner [72]. Thus, the interaction of symbiotic organisms within lichens continues to be a fascinating topic. To identify the photobiont of Antarctic *C. borealis*, we carried out amplicon sequencing of two field samples using the algal-specific ITS region (Table 2, Table S3). The total sequencing reads were over 390 Mb in length, and the number of reads was 1,302,402 and 1,327,794, respectively. Approximately 70% of the total sequencing reads were successfully connected to 453,505 and 480,246 read pairs, and after removing low-quality sequences, the final clustered reads were 53.1 Mb and 49.5 Mb in length (Table S3). For multiple alignments and BLAST search analyses, 98.9–99% of the reads were matched to *Asterochloris irregularis* (NCBI accession AM906000) with over 98% identity. Although the remaining reads—comprising *Coccomyxa antarctica*, *Trebouxia irregularis*, *Trebouxia jamesii*, and *Trebouxia impressa* from Trebouxiophyceae, and *Coenochloris signiensis* and uncultured Chlorophyta taxa from Chlorophyceae—constituted less than 0.1%, these accessory algae are worth exploring further (Table 2).

The genus *Asterochloris* is composed of a photobiont of more than 20 lichen genera, predominantly *Cladonia*, *Lepraria*, and *Stereocaulon*, in diverse habitats [73,74,75,76,77,78,79,80,81,82]. Recently, *A. irregularis* was reported to be a photobiont of *Cladonia arbuscula*, *Stereocaulon pileatum*, *Stereocaulon subcoralloides*, and *Stereocaulon botryosum* in Central and Eastern Europe, and was suggested to be a discriminated species based on its molecular phylogeny of combined ITS and actin 1 sequences, its chloroplast morphology, and its morphological features during cell division [80]. In this study, we report that *A. irregularis* is the major photobiont of Antarctic *C. borealis*, and its photosynthetic performance is downregulated during daytime in its natural habitat. In addition, the NPQ reactions of *C. borealis* were induced by daytime light intensity and desiccating conditions. We surmise that such photosynthetic performance is derived from the photobionts, but how such physiological responses are regulated between isolated photobionts and fungal partners should be carefully examined. In a study of *C. vulcani*, for example, the photoprotective response to desiccation and irradiation stress was further enhanced in the lichen form compared to either the isolated mycobiont or phycobiont. During 9 weeks of desiccation, the lichen form maintained photosynthetic pigments, including chlorophyll and xanthophyll, together with the antioxidant α-tocopherol, which completely recused them after rehydration. However, the isolated alga failed to do so. Such phenotypes highlight that biochemical interactions between the fungal and algal partners are essential to resist oxidative and high light stress in nature [21]. Therefore, comparison of the physiological responses between the lichen *C. borealis* and its photobionts is necessary to elucidate the regulatory pathways of resistance to desiccation and light stress. 

## 3. Materials and Methods

### 3.1. Microclimate and Chlorophyll Fluorescence Measurement in the Field

Field observation was carried out from 23–30 January 2019 at the KGL01 site (KGL01: 62°14′24″ S, 58°44′36″ W), Barton Peninsula, King George Island. Three thalli of each *C. borealis* and *Usnea* sp. were selected for chlorophyll fluorescence measurements. Any moss was carefully removed to minimize fluorescence interference. Chlorophyll fluorescence monitoring was performed using MONI-PAM (MONITORING-PAM Multi-Channel Chlorophyll Fluorometer, Walz, Effeltrich, Germany), which was composed of a data acquisition system (MONI-DA) and six emitter-detector units (MONI-head/485) connected to HEX-PAM. The operation method and its application were derived from those published by Porcar-Castella et al. [83]. A blue LED (emission maximum: 455 nm ± 9 nm) was used as the light source for actinic light, saturating pulse, and measuring light. In this study, the light intensity for measuring light was 0.9 μmol/m^2^/s and the saturating pulse was approximately 2500 μmol/m^2^/s with a duration of up to 2 s. To reduce the actinic light effect on samples, the lights were turned off between measuring points and automatically turned on a few seconds before the saturating pulse analysis. Fluorescence (*F* and *Fm*’) was detected before and after a saturating pulse every 20 min. At the same time, the PPFD and temperature were recorded. 

There were six MONI-heads fixed to the lichen samples, three for the *Usnea* sp. and three for *C. borealis*, at a 120° angle from the horizontal axis, but two failed to retrieve data. Volumetric soil moisture was measured at a depth of 10 cm using a Soil Moisture Smart Sensor (#EC-5, Decagon Devices, Pullman, WA, USA) and recorded by a HOBO Micro Station Data Logger (#H21-002, Onset, Bourne, MA, USA) every 10 min. Rainfall records, assessed every 10 min, were acquired from the AWS managed by the Climate Research Group at King Sejong Station.

### 3.2. Genomic DNA Extraction and Lichen Identification

*C. borealis* samples, with a similar size and weight (without podetia), were collected from the KGL01 site and transferred to the laboratory at King Sejong Station. Two thalli were used for total genomic DNA extraction. The samples were washed twice with 70% ethanol and 0.1% Tween 20 and rinsed with distilled water (DW). The dried sample was ground with liquid nitrogen, and total genomic DNA was extracted by the cetyltrimethylammonium bromide (CTAB) method [84]. Total genomic DNA was used for both lichen identification and amplicon sequencing of the photobiont. For lichen identification, the ITS region was amplified from the prepared total genomic DNA (#1 and #2) using fungal-specific ITS1 and ITS4 primers [85]. The purified PCR product was used for sequencing analysis (Bionics, Seoul, Korea) and the sequencing results were used for BLAST searches in NCBI. 

### 3.3. Desiccation and Rehydration Treatment

The air-drying experiment was performed using field samples in a laboratory at King Sejong Station, Barton Peninsula. DW with an ion conductivity of 10 μS/cm (pH 7.8) was used for rehydration. A preliminary experiment confirmed that the water content of *C. borealis* thalli reaches full rehydration in 10 min, when its total weight no longer increases (data not shown). Based on this result, we used the following method to rehydrate the thalli: spray with distilled water for 10 min and then remove excess water from the thalli using Kimwipes (Kimberly-Clark Professional, Roswell, GA, USA). Before air-drying, 10 individual samples were fully rehydrated as described above. The air-drying experiment was performed under the following laboratory conditions: air humidity was 50%, temperature was 16 °C, and light intensity was 50 μmol/m^2^/s. Thallus weight and *Fv*’/*Fm*’ were measured every 10 min using the MINI-PAM-II fluorometer (Walz) until there was no change in weight, which required approximately 3 h. After an additional 21 h of air-drying, fluorescence was no longer detected. However, after 10 min of rehydration with the water spray, fluorescence was detected. This rehydrated state was maintained for 3 h and the fluorescence was measured. The RWC of the sample during dehydration was measured using an analytical balance (#PG503-S, METTER TOLEDO, Greifensee, Switzerland) to a precision of 0.1 mg. The RWC was calculated as described by Sun [86]. 

The RLC experiment under different light intensities and desiccation conditions was carried out at the laboratory of the Korea Polar Research Institute (Incheon, Korea) using IMAGING-PAM (Walz). The samples were stabilized at 50 μmol/m^2^/s light with an 18:6 light:dark cycle at 8 °C with hydration every three days in a growth chamber. To reproduce the microclimate conditions of the species’ natural habitat (Figure 2), we established two light intensities—50 and 220 μmol/m^2^/s—based on the average light intensity at dawn (04:00–06:00) and daytime (06:00–22:00), respectively. These average light intensities were calculated from >50% of the Y(II) value to exclude conditions that damage photosynthetic activity. In addition, the temperature was fixed at 8 °C to minimize low-temperature stress conditions. We chose mild and severe desiccation conditions of 85% RH for SD and 5% RH for RD, respectively. SD and RD were prepared in a humid box (20 cm × 15 cm × 20 cm) with 20 mL of DW (RH ~85%) or silica gel (RH < 5%), respectively. The samples were placed on a mesh plate 10 cm from the bottom for the given desiccation treatment. Ten individual samples were used for each SD and RD treatment after 2 h of light activation at 50 μmol/m^2^/s. Rehydration after desiccation treatment for 24 h was conducted with a water spray, and excess water was removed before the rehydrated samples were transferred to the SD container (RH 85%). Desiccation and rehydration treatments for the RLC experiment were performed using ten biological replicates for each condition. A total of forty thalli was used for four conditions (two light intensities x two desiccation conditions). Experiments for SD and RD treatments were repeated two and three times, respectively. After desiccation-rehydration treatment, the samples were re-stabilized at 50 μmol/m^2^/s light with an 18:6 light:dark cycle at 8 °C with hydration for a week.

### 3.4. Chlorophyll Fluorescence Measurement in the Laboratory

For the RLC experiment, chlorophyll fluorescence was measured using IMAGING-PAM (Walz,) during desiccation-rehydration treatment. After 6 h in the dark, minimum chlorophyll fluorescence (*Fo*) and maximum chlorophyll fluorescence (*Fm*) elicited by a strong saturation pulse were measured and then used to calculate the maximal photochemical quantum yield (*Fv*/*Fm*): *Fv*/*Fm* = (*Fm* − *Fo*)/*Fm* [41]. The average *Fv*/*Fm* value of *C. borealis* samples stored in the dark was ~0.62 (± 0.03). Desiccation and rehydration treatment was conducted under the same light cycle (18:6 light:dark), which began after 2 h of light activation. The effective PSII quantum yield (YII) or *Fv*’/*Fm*’ = (*Fm*’ − *F*)/*Fm*’ was calculated from the light-activated samples [41]. The *Fm*’ value represents the maximum fluorescence yield in a light-adapted sample, while the *F*’ value represents the level of fluorescence shortly before the application of the saturating pulse [41]. The RLC reveals the saturation features of electron transport and the overall photosynthetic performance in plants [58]. The RLC experiment for the desiccation-rehydration cycle was performed under 50 μmol/m^2^/s (dawn light) and 220 μmol/m^2^/s (daylight) conditions every 30 min. The coefficient of photochemical fluorescence quenching, qP, was calculated as (*Fm*’ − *F*)/(*Fm*’ − *Fo*) [61,62]. The quantum yield of non-light induced non-photochemical fluorescence quenching, Y(NO) was calculated as 1/[NPQ + 1 + qL × (*Fm*/*Fo* − 1)] [60]. The quantum yield of light-induced NPQ was calculated as Y(NPQ) = 1 − Y(II) − Y(NO) [60]. The curve fitting method of Ralph and Gademann was utilized [58]. 

### 3.5. High-Throughput Amplicon Sequencing for Photobiont Diversity

Total genomic DNA from two *C. borealis* samples was used to amplify the algal ITS2 region with 5.8SF (5′-CGG ATA TCT TGG CTC TCG CA-3′) and LSU0012_R (5′-AGT TCA GCG GGT CTT G-3′) primers [79] in conjunction with adapter sequences of the Illumina Miseq platform. The first PCR was conducted with a 15-μL reaction volume containing 3 μL of 5× Phusion HF Buffer, 1.5 μL of 2 mM dNTPs, 1.5 μL of primer mix (10 μM), 1.5 μL of 3 M betaine, 1 μL of template DNA, 0.1 μL of Phusion DNA polymerase, and 6.4 μL DW. The PCR cycle conditions included initial denaturation at 98 °C for 30 s, followed by 20 cycles of denaturation at 98 °C for 30 s, annealing at 60 °C for 1 min, and extension at 72 °C for 15 s, with a final extension at 72 °C for 5 min. The PCR product was confirmed on a 1% agarose gel stained with GelRed and the remaining primers and dNTPs were removed using ExoSAP-IT PCR product cleanup reagent (#78200, ThermoScientific, Seoul, Korea). The purified PCR product was used as a template for secondary PCR for conjugation with the index sequences (i5 and i7). The secondary PCR was performed in a 20-μL reaction volume containing 4 μL of 5× Phusion HF Buffer, 2 μL of 2 mM dNTPs, 2 μL of 3 M betaine, 2 μL of primer mix (5 μM), 3 μL of purified PCR product, 0.2 μL of Phusion DNA polymerase, and 6.8 μL DW. The PCR cycle conditions included initial denaturation at 98 °C for 30 s, followed by 25 cycles of denaturation at 98 °C for 30 s, annealing at 60 °C for 15 s, and extension at 72 °C for 15 s, with a final extension at 72 °C for 5 min. The PCR product was quantified and the clean-up reaction was repeated. 

Paired-end (PE) reads averaging 301 bp were produced by the Illumina MiSeq platform and merged into connected sequences of 379–383 bp by joining both ends using clc_assembly_cell version 4.3 (https://www.qiagenbioinformatics.com/products/clc-assembly-cell/). Low-quality sequences were removed from the connected sequences using Trimmomatic version 0.38 (http://www.usadellab.org/cms/?page=trimmomatic) with the following parameters: minimum quality, 20 and minimum length, 50. The high-quality connected sequences were clustered based on the criterion of 100% homology using CD-HIT version 4.7 (http://weizhongli-lab.org/cd-hit/). Thereafter, representative sequences and their copy number information were obtained. Representative sequences were used as nucleotide queries for similarity searches. Similarity searches for nucleotide and peptide queries were performed against the NCBI non-redundant nucleotide database using BLAST version 2.7.1+ (https://ncbiinsights.ncbi.nlm.nih.gov/2017/10/27/blast-2-7-1-now-available/) with a cutoff *e*-value of 1 × 10^−4^. Hit subject sequences with >98% identity were further selected from the similarity results; their NCBI taxonomy information was extracted and used for taxonomical classification of the query sequences. 

### 3.6. Statistical Analysis

For the data shown in Figure 3, the linear fitting analysis between Y(II) and the three microclimate parameters was performed using Origin 8.5 (OriginLab Corp., Northampton, MA, USA). The slope, standard error (SE), and R^2^ values were calculated from this linear fitting analysis. In Figure 5, the mean value was calculated by two-way ANOVA, and mean comparison among the data was analyzed based on the Tukey’s honestly significant difference (HSD) test. Significant differences among treatments were denoted with different letters at *p* < 0.05 level. This statistical analysis was performed using SigmaPlot 11 software (Systat Software Inc., San Jose, CA, USA).

## Figures and Tables

**Table 1:** Mean photosynthetic efficiency Y(II), PPFD, and temperature changes during dawn, daytime, and night. Numbers in parentheses indicate ± standard deviation.

	Night	Dawn	Daytime
Time range	22:00–4:00	4:00–6:00	6:00–22:00
Total data points	133	42	316
PPFD (μmol/m^2^/s)	4 (± 2)	47 (± 37)	357 (± 289)
Temperature (°C)	1.5 (± 1.3)	1.5 (± 1.5)	4.7 (± 2.9)
*C. borealis*			
Y(II)	0.41 (± 0.16)	0.31 (± 0.12)	0.26 (± 0.15)
Inactivation (%) *	7.1%	7.1 %	14.1%
*Usnea* sp.			
Y(II)	0.09 (± 0.23)	0.06 (± 0.19)	0.19 (± 0.25)
Inactivation (%)	87.6%	91.7%	58.9%

* Percent inactivation indicates the proportion of Y(II) = 0 in the total data points, because there is no difference between the *F* and *Fm*’ values.

**Table 2:** Photobiont species list of two Antarctic *Cladonia borealis* samples analyzed by amplicon sequencing of the ITS region.

Id	NCBI Accession	Species	ANT#1		ANT#2	
			Copy No.	%	Copy No.	%
OTU01	AM906000	*Asterochloris irregularis*	389,307	98.994	358,306	99.051
OTU02	MH415413	*Asterochloris irregularis* voucher VancurovaO5	1902	0.484	56	0.015
OTU03	MF465900	*Coccomyxa antarctica* isolate FACHB-2140	475	0.121	183	0.051
OTU04	FJ626732	*Trebouxia irregularis* strain SAG 33.85	367	0.093	367	0.101
OTU05	HQ404871	*Coenochloris signiensis* strain CCCryo 135-01	108	0.027	14	0.004
OTU06	KX147269	*Trebouxia jamesii* isolate O2131C0001ASBM100076	104	0.026	424	0.117
OTU07	KY559180	*Trebouxia* sp. ‘vagua’ isolate IB97	103	0.026	53	0.015
OTU08	KX181276	*Trebouxia impressa* isolate L2239p	95	0.024	10	0.003
OTU09	JX435372	Uncultured Chlorophyta clone ALCE9	94	0.024	59	0.016
OTU10	JX435341	Uncultured Chlorophyta clone ALBA5	84	0.021	176	0.049

## Supplementary Materials

The following are available online at https://www.mdpi.com/2223-7747/9/1/85/s1, Figure S1: Monthly changes in microclimate factors at the study site, KGL01, in Barton Peninsula, Antarctica, Figure S2: Diurnal dynamics of (a) PPFD (μmol/m^2^/s), (b) temperature (°C), (c) volumetric soil moisture (%), (d) the Y(II) of *C. borealis* and (e) the Y(II) of *Usnea* sp, Figure S3: Plots of Y(II) with three microclimate factors recorded during all period of field observation, Figure S4: Air-drying of field *C. borealis* samples with changes of (a) RWC (%), (b) the Y(II) value, and (c) the relative recovery rate of Y(II) after rehydration, Figure S5: The rapid light curves of *C. borealis* by desiccation and rehydration treatment under different light intensity, dawn-light (50 μmol/m^2^/s) and daytime-light (220 μmol/m^2^/s), Figure S6: Changes of Y(II), Y(NPQ) and 1-qP of *C. borealis* during rapid light curve experiment under the slow desiccation (SD), rapid desiccation (RD) and rehydration after 24hr-RD treatment (RD→Rehyd.) with different light intensity, dawn-light (50 μmol/m^2^/s) and daytime-light (220 μmol/m^2^/s), Table S1: The mean, min and max values of Y(II) of *C. borealis* and the microclimates, Table S2: Linear regression of Y(II) with three microclimate factors during all period of field observation, Table S3: Results of high-throughput amplicon sequencing output amplifying the algal specific ITS region from two samples of *C. borealis*.

## Abbreviations

Y(II)	Quantum efficiency of photosystem II
Y(NO)	Quantum yield of nonregulated and non-photochemical energy dissipation at photosystem II
Y(NPQ)	Quantum yield of non-photochemical quenching at photosystem II
NPQ	non-photochemical quenching
ETR	Electron transfer rate
PSII	Photosystem II
PSI	Photosystem I
OEC	Oxygen evolving center
PQ	Plastoquinone
ROS	Reactive oxygen species
PPFD	Photosynthetic photon flux density
RH	Relative humidity
RWC	Relative water content
RD	Rapid desiccation
SD	Slow desiccation
RLC	Rapid light curve
AWS	Automatic weather station
