# Supplementary material for: Study of Ecophysiological Responses of the Antarctic Fruticose Lichen Cladonia borealis Using the PAM Fluorescence System under Natural and Laboratory Conditions
Source: Plants (Basel). 2020 Jan 9;9(1):85. doi: 10.3390/plants9010085 (PMC7020452; doi:10.3390/plants9010085)
Supplement: Supplementary file 1 [file plants-09-00085-s001.pdf]

# Supplementary materials

**Article title:** Study of Ecophysiological Responses of the Antarctic Fruticose Lichen *Cladonia borealis* Using the PAM Fluorescence System under Natural and Laboratory Conditions

**Author:** Sung Mi Cho [1], Hyoungseok Lee [2,3], Soon Gyu Hong [2], and Jungeun Lee [1*]

[1] Unit of Research for Practical Application, Korea Polar Research Institute, Incheon 21990, South Korea; smcho@kopri.re.kr (S.M.C.)

[2] Division of Polar Life Sciences, Korea Polar Research Institute, Incheon 21990, South Korea; soulaid@kopri.re.kr (H.L.); polypore@kopri.re.kr (S.G.H.)

[3] Polar Sciences, University of Science and Technology, Daejeon 34114, South Korea

* Correspondence: jelee@kopri.re.kr; Tel.: +82-32-760-5576 (J.L.)

## List of supplementary materials

**Table S1**. The mean, min and max values of Y(II) of *C. borealis* and the microclimates.

**Table S2**. Linear regression of Y(II) with three microclimate factors during all period of field observation.

**Table S3**. Results of high-throughput amplicon sequencing output amplifying the algal specific ITS region from two samples of *C. borealis*.

**Figure S1**. Monthly changes in microclimate factors at the study site, KGL01, in Barton Peninsula, Antarctica.

**Figure S2**. Diurnal dynamics of (a) PPFD (μmol/m$^2$/s), (b) temperature (°C), (c) volumetric soil moisture (%), (d) the Y(II) of *C. borealis* and (e) the Y(II) of *Usnea* sp.

**Figure S3**. Plots of Y(II) with three microclimate factors recorded during all period of field observation.

**Figure S4**. Air-drying of field *C. borealis* samples with changes of (a) RWC (%), (b) the Y(II) value, and (c) the relative recovery rate of Y(II) after rehydration.

**Figure S5**. The rapid light curves of *C. borealis* by desiccation and rehydration treatment under different light intensity, dawn-light (50 μmol/m$^2$/s) and daytime-light (220 μmol/m$^2$/s).

**Figure S6**. Changes of Y(II), Y(NPQ) and 1-qP of *C. borealis* during rapid light curve experiment under the slow desiccation (SD), rapid desiccation (RD) and rehydration after 24hr-RD treatment (RD→Rehyd.) with different light intensity, dawn-light (50 μmol/m$^2$/s) and daytime-light (220 μmol/m$^2$/s).

**Table S1**. The mean, min and max values of Y(II) of *C. borealis* and the microclimates. These data were excluded night time. Characters A, B, and C in parentheses indicates Region in Figure 2.

| Date | | Y(II) | PPFD | Temp. | Soil moisture | Air RH | Rainfall* |
|---|---|---|---|---|---|---|---|
| | | | μmol/m²/s | °C | % | % | mm |
| 23th | Mean | 0.335 | 323 | 3.37 | 0.213 | 90.34 | 2 |
| | SD | (±0.044) | (±260) | (±2.47) | (±0.004) | (±3.04) | |
| | Min | 0.263 | 10 | 0.10 | 0.206 | 83.50 | |
| | Max | 0.433 | 730 | 8.20 | 0.219 | 94.20 | |
| 24th | Mean | 0.287 | 319 | 4.41 | 0.233 | 87.11 | 60 |
| (A) | SD | (±0.061) | (±300) | (±1.97) | (±0.011) | (±1.36) | |
| | Min | 0.190 | 5 | 0.10 | 0.202 | 84.60 | |
| | Max | 0.480 | 1295 | 8.80 | 0.247 | 90.40 | |
| 25th | Mean | 0.288 | 355 | 0.92 | 0.231 | 78.27 | 18 |
| | SD | (±0.033) | (±275) | (±1.38) | (±0.007) | (±5.64) | |
| | Min | 0.241 | 5 | -1.10 | 0.217 | 69.94 | |
| | Max | 0.362 | 1040 | 4.30 | 0.239 | 90.70 | |
| 26th | Mean | 0.189 | 342 | 4.32 | 0.208 | 82.96 | 0 |
| (B) | SD | (±0.042) | (±256) | (±3.06) | (±0.004) | (±3.16) | |
| | Min | 0.115 | 4 | -0.60 | 0.203 | 77.41 | |
| | Max | 0.294 | 1146 | 10.50 | 0.216 | 90.80 | |
| 27th | Mean | 0.205 | 300 | 4.77 | 0.204 | 89.31 | 9 |
| (C) | SD | (±0.069) | (±210) | (±1.51) | (±0.003) | (±4.00) | |
| | Min | 0.114 | 4 | 2.10 | 0.199 | 81.20 | |
| | Max | 0.447 | 641 | 7.90 | 0.214 | 95.10 | |
| 28th | Mean | 0.202 | 466 | 5.99 | 0.204 | 90.06 | 1 |
| | SD | (±0.043) | (±447) | (±3.37) | (±0.006) | (±5.66) | |
| | Min | 0.100 | 3 | 2.20 | 0.195 | 80.10 | |
| | Max | 0.284 | 1545 | 14.10 | 0.212 | 96.60 | |

* This is the sum of accumulated rainfall during one day.

**Table S2.** Linear regression of Y(II) with three microclimate factors during all period of field observation. These data was calculated from Figure S2.

| | | *C. borealis* | | *Usnea* sp. | |
|---|---|---|---|---|---|
| Y(II) vs. | | Slop (SE) | $R^2$ | Slop (SE) | $R^2$ |
| PPFD | #1 | -2.163E-4 (1.53E-5) | 0.344 | -3.50E-4 (2.92E-5) | 0.504* |
| | #2 | -2.068E-4 (1.76E-5) | 0.217 | -6.27E-4 (3.22E-5) | 0.713* |
| Temperature | #1 | -0.013 (0.002) | 0.104 | -0.033 (0.004) | 0.255 |
| | #2 | -0.023 (0.001) | 0.261 | -0.060 (0.004) | 0.555* |
| Soil moisture | #1 | -0.884 (0.400) | 0.010 | 0.837 (0.999) | -0.002 |
| | #2 | 1.664 (0.440) | 0.026 | -1.946 (1.221) | 0.010 |

**Table S3**. Results of high-throughput amplicon sequencing output amplifying the algal specific ITS region from two samples of *C. borealis*.

| | Total reads (Sequencing) | | Joined pairs | | Total reads (Clustered) | |
|---|---|---|---|---|---|---|
| | Length (bp) | No. | No. | % | Length (bp) | No. |
| #1 | 392,023,002 | 1,302,402 | 453,505 | 69.64 | 53,111,651 | 423,923 |
| #2 | 399,665,994 | 1,327,794 | 480,246 | 72.34 | 49,578,246 | 396,086 |

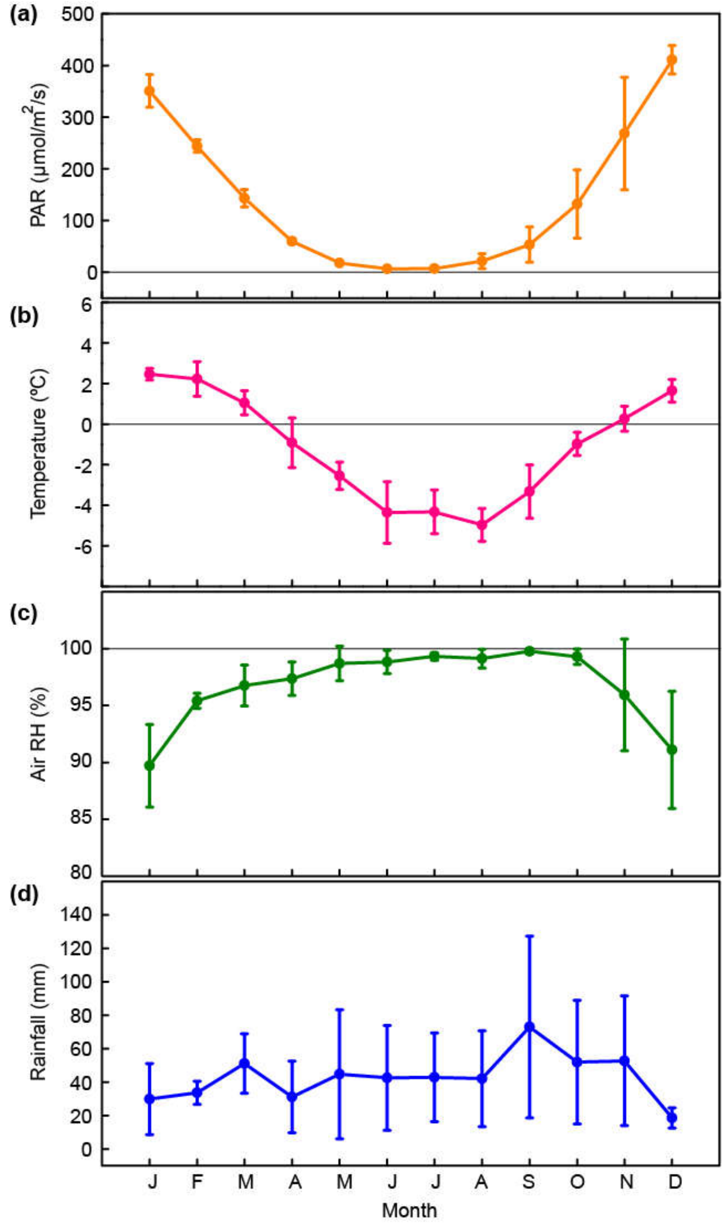


**Figure S1**. Monthly changes in microclimate factors at the study site, KGL01, in Barton Peninsula, Antarctica. The data are presented as the monthly average values of the data obtained from the HOBO loggers installed on the site (2013 to 2018 years), except for the rainfall data (2014 to 2017 years). Error bars indicate standard deviations.

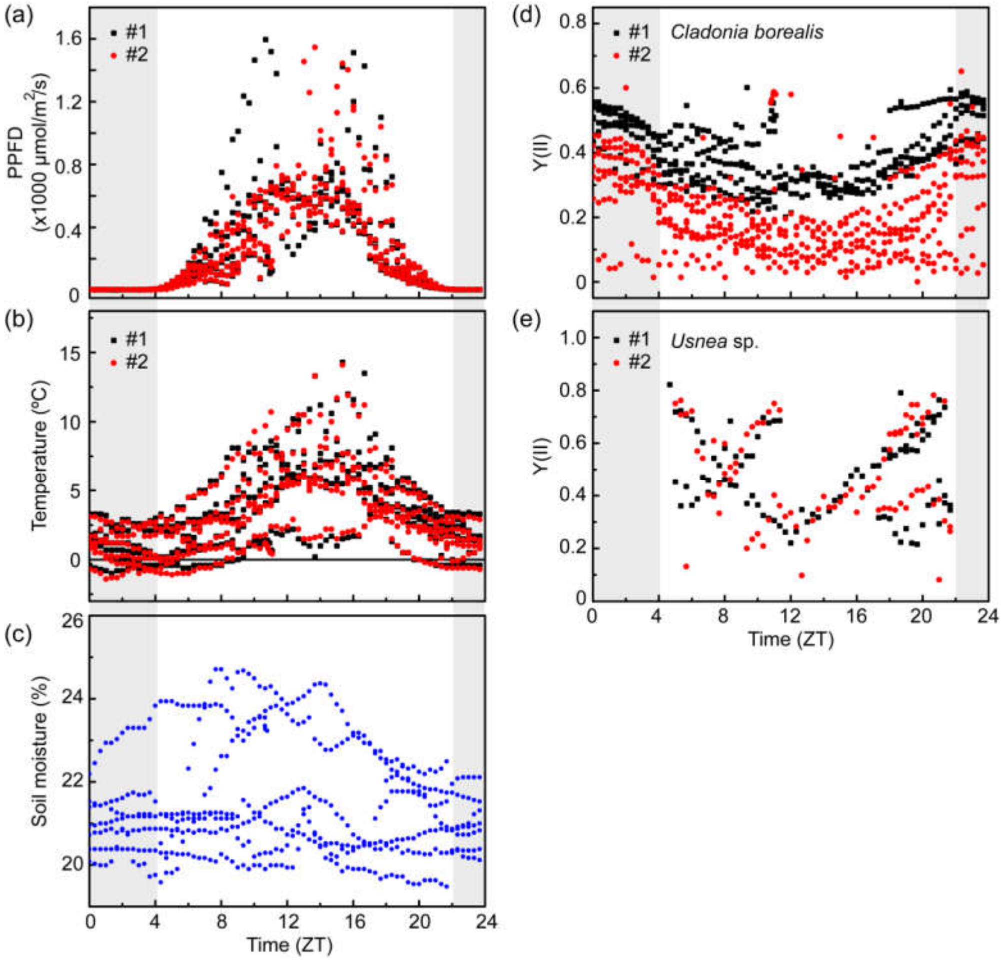


**Figure S2**. Diurnal dynamics of (a) PPFD (μmol/m²/s), (b) temperature (°C), (c) volumetric soil moisture (%), (d) the Y(II) of *C. borealis* and (e) the Y(II) of *Usnea* sp. These data were prepared from the observation during 22nd-30th of January in 2019. The grey shading indicates the night time (from 10 PM to 4 AM) defined by less than 10 μmol/m²/s of PPFD. Black and red symbols indicate the sample #1 and #2, respectively.

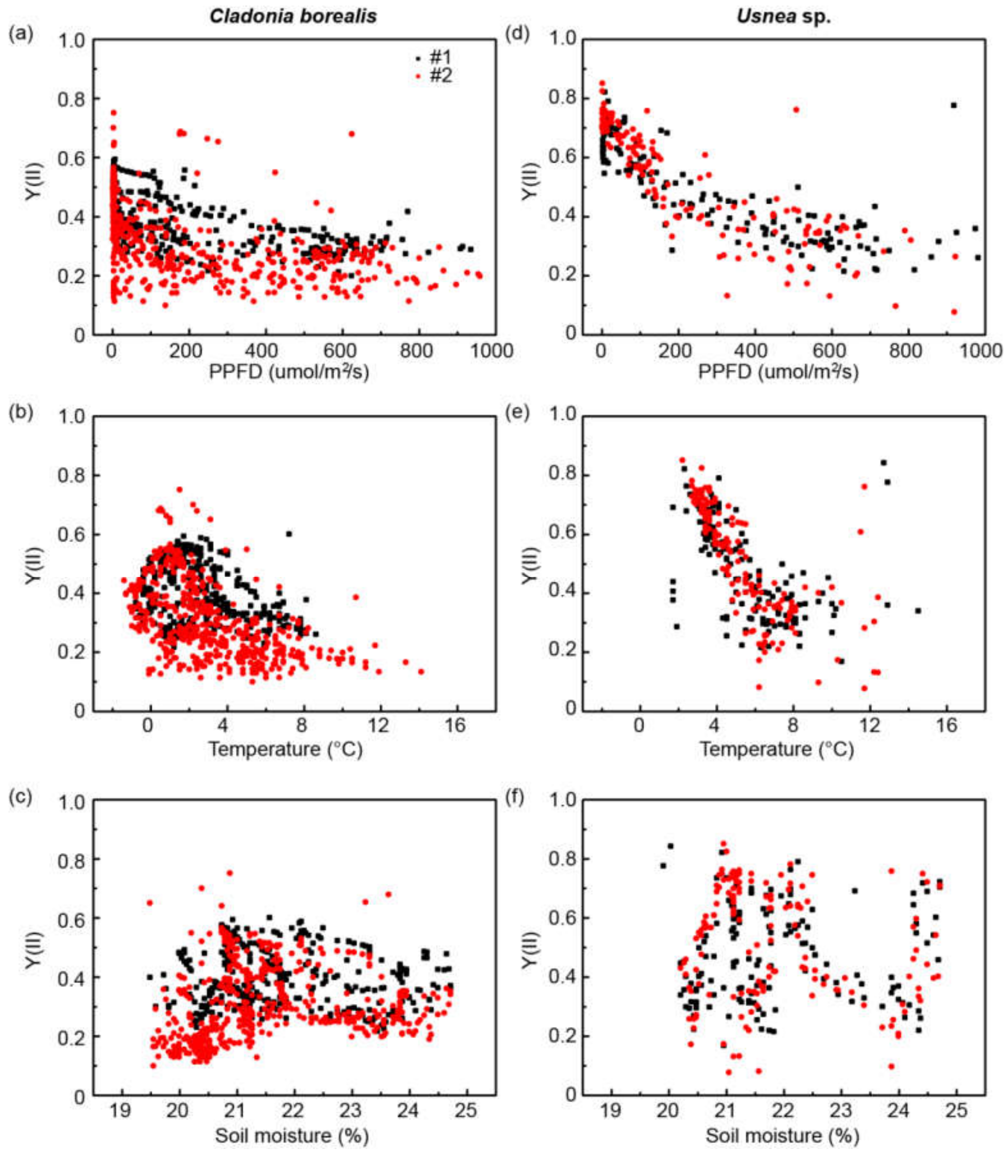


**Figure S3**. Plots of Y(II) with three microclimate factors recorded during all period of field observation. (a-c) Data from two samples of *Cladonia borealis* (d-f) Data from two samples of *Usnea* sp. Black and red symbols indicate the sample #1 and #2, respectively.

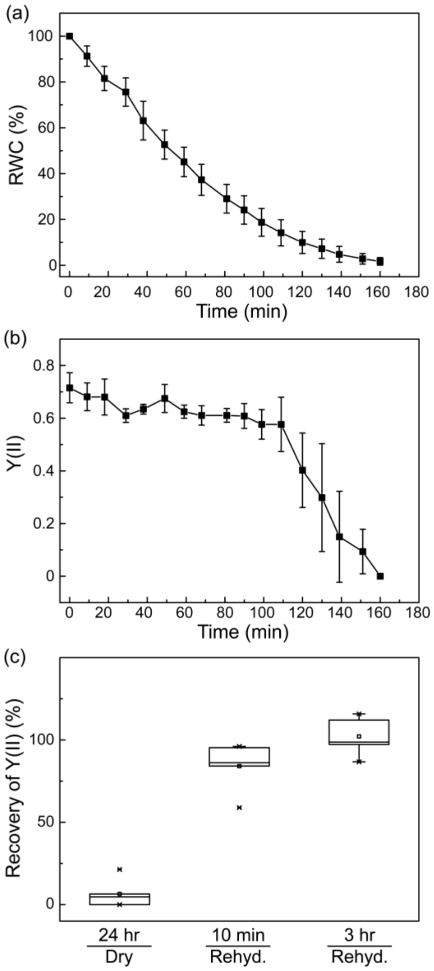


**Figure S4**. Air-drying of field *C. borealis* samples with changes of (a) RWC (%), (b) the Y(II) value, and (c) the relative recovery rate of Y(II) after rehydration. Experiment was performed using ten biological replicates ($n = 10$) and the average with standard deviations were shown.

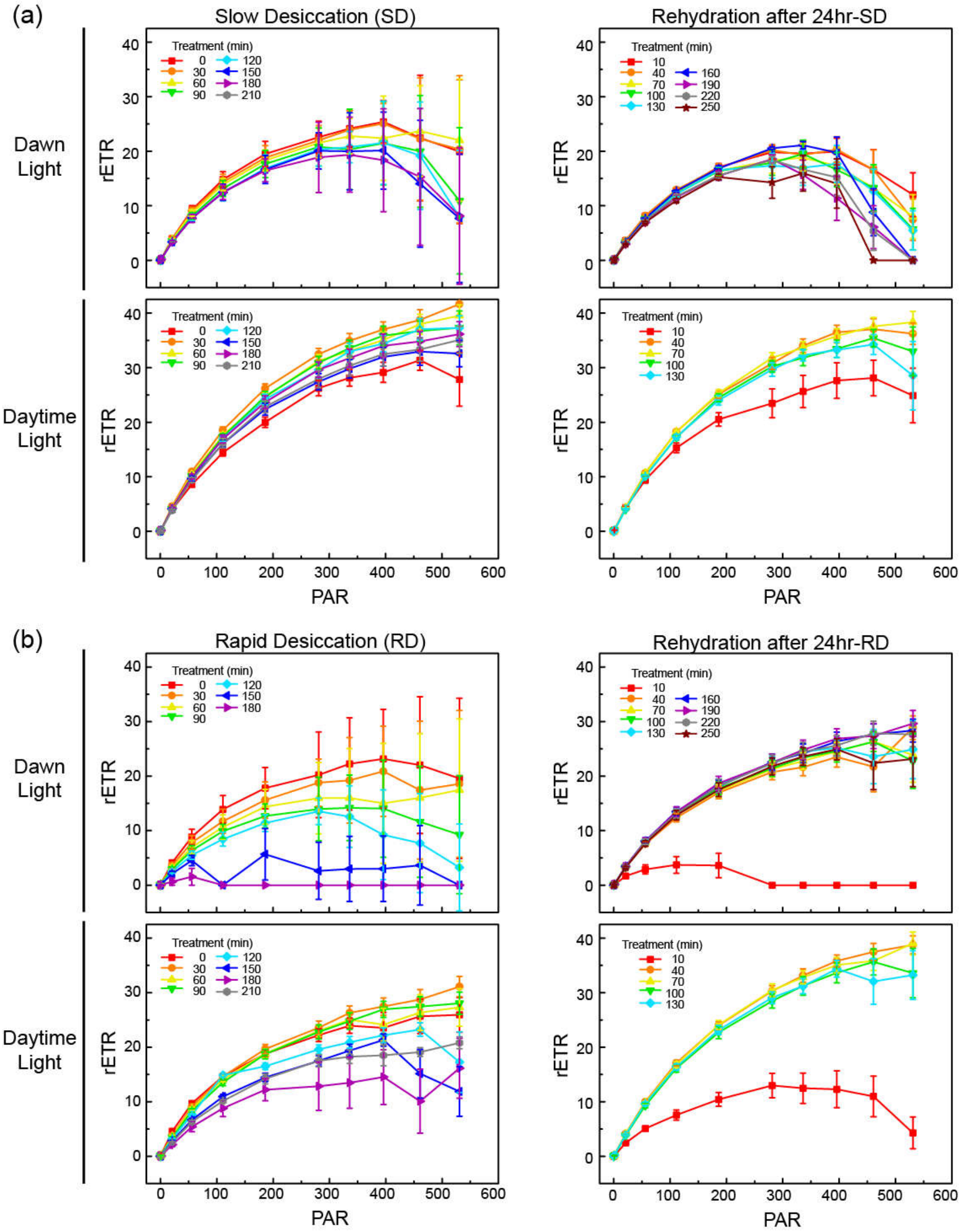


**Figure S5**. The rapid light curves of *C. borealis* by desiccation and rehydration treatment under different light intensity, Dawn (50 $\mu$mol/m²/s) and Daytime (220 $\mu$mol/m²/s). (a) Slow desiccation (SD) and rehydration after 24hr-SD. (b) Rapid desiccation (RD) and rehydration after 24hr-RD. Each value indicates the average score ± standard deviation. The ten biological replicates were used for each treatment ($n$ = 10). Experiments were repeated at least two or three times using the same thalli after re-stabilizing at 50 μmol/m²/s light with an 18:6 light:dark cycle at 8 °C with hydration for a week.

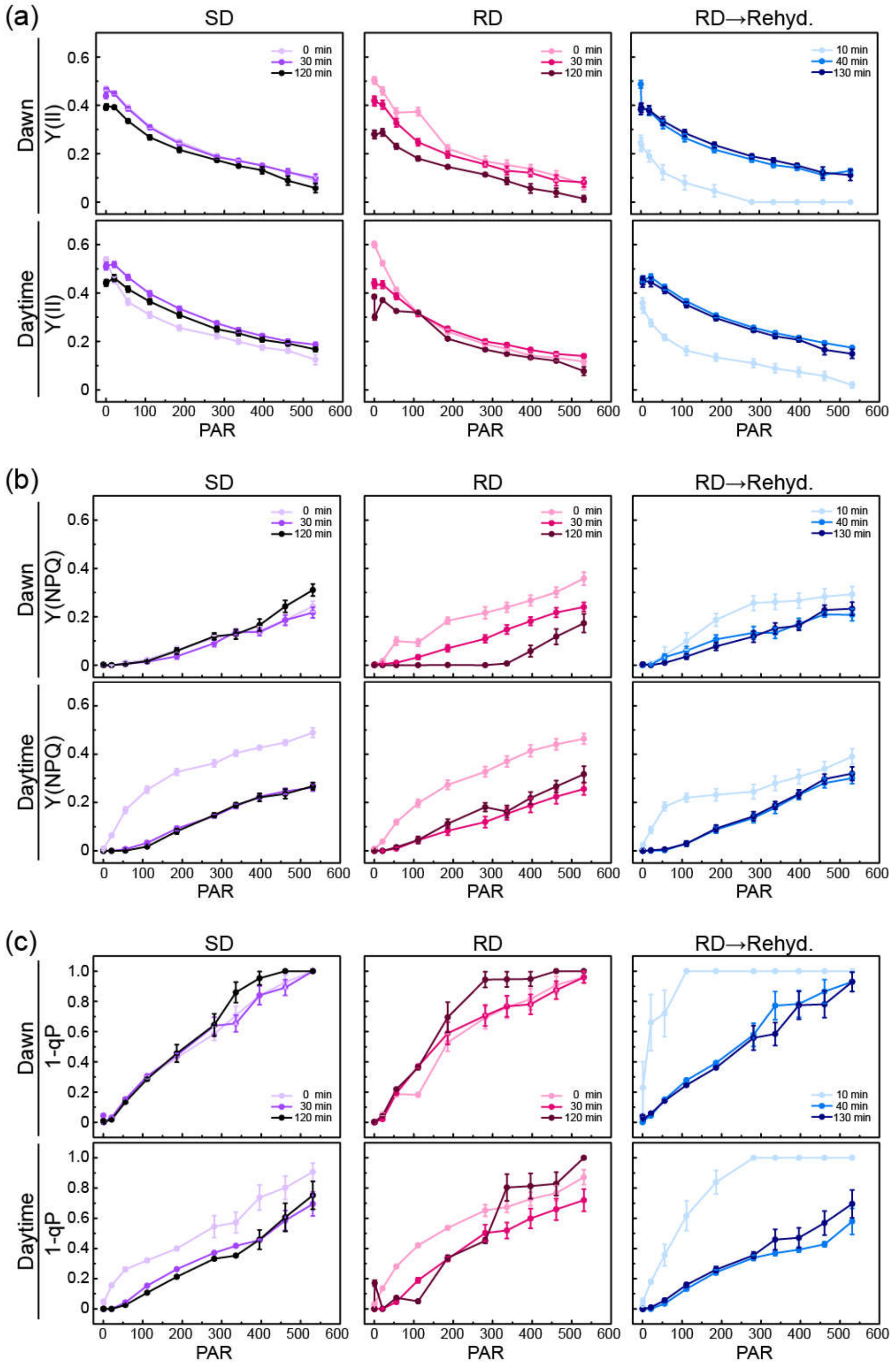


**Figure S6**. Changes of Y(II), Y(NPQ) and 1-qP of *C. borealis* during rapid light curve experiment under the slow desiccation (SD), rapid desiccation (RD) and rehydration after 24hr-RD treatment (RD→Rehyd.) with different light intensity, dawn (50 $\mu$mol/m²/s) and daytime (220 $\mu$mol/m²/s). Each value indicates the average score ± standard deviation. The ten biological replicates were used for each treatment ($n$ = 10). Experiments were repeated at least two or three times using the same thalli after re-stabilizing at 50 μmol/m²/s light with an 18:6 light:dark cycle at 8 °C with hydration for a week.
